# Editorial: Intraoperative Ultrasound in Brain Tumor Surgery: State-Of-The-Art and Future Perspectives

**DOI:** 10.3389/fonc.2021.780517

**Published:** 2021-11-02

**Authors:** Massimiliano Del Bene, Francesco DiMeco, Geirmund Unsgård

**Affiliations:** ^1^ Department of Neurosurgery, Fondazione Istituto di Ricovero e Cura a Carattere Scientifico (IRCCS) Istituto Neurologico Carlo Besta, Milan, Italy; ^2^ Department of Experimental Oncology, European Institute of Oncology (IEO), Istituto di Ricovero e Cura a Carattere Scientifico (IRCCS), Milan, Italy; ^3^ Department of Pathophysiology and Transplantation, University of Milan, Milan, Italy; ^4^ Department of Neurological Surgery, Johns Hopkins Medical School, Baltimore, MD, United States; ^5^ Norwegian University of Science and Technology, Trondheim, Norway

**Keywords:** intra-operative ultrasound, neurosurgery, oncology, brain tumour, advanced ultrasound

Ultrasound (US) is undoubtedly the most versatile imaging modality in medicine. It can provide real-time, high-resolution images in almost every setting, (e.g. ward, emergency room, outpatients clinic, operating room, etc) and in almost every anatomical region (e.g. abdomen, thorax, limbs, intra-cranial, etc). The central nervous system, owing to its optimal mechanical/acoustic properties, represents the ideal substrate for US propagation and image generation (1). The earliest reports on intraoperative ultrasound (ioUS) in neurosurgery date back to the late 1970s (2). Since this time, US applications have been described both in brain and spine surgery. Intriguingly, after initial enthusiasm, ioUS has mainly been confined to marginal applications because of several intrinsic limitations such as poor resolution, user dependency, difficult semiology/orientation, and lack of specific training (1, 3). More recently, the introduction of major technical improvements has gradually framed ioUS as one of the most versatile and valuable techniques for intra-operative imaging in neurosurgery even if it is still under utilized, mainly because of the lack of specific training.

The multi-modal nature of ioUS can guide surgery, offering both structural and functional information in real-time. The classical B-mode US has reached an exceptional spatial and temporal resolution, providing mainly structural information (4–6). In the effort to overcome B-mode limitations [e.g. specific semiotics, orientation (5, 6)], fusion imaging has been introduced, which enables practitioners to match the ioUS images to the pre-operative magnetic resonance imaging (MRI) to better understand semiotics, facilitate orientation, and compensate brain shift occurrence (7–10). Doppler imaging (color-, spectral-, power-, micro-vessels) permits the study of blood flow and at the same time, the anatomy of the vessels (8, 11, 12), providing valuable information for both vascular and oncological neurosurgery (8, 12–15). Further evolution of Doppler imaging has been enabled by contrast-enhanced ultrasound (CEUS), which relies on purely endovascular contrast agents, namely gas-filled micro-bubbles, to provide a dynamic and continuous representation of the vascular tree, from a structural and a functional standpoint, and of the tissue perfusion pattern (16–18). Its applications, in oncological surgery, encompass tumor identification and characterization, assessment of the pattern of vascularization, and the identification of residual tumours (12, 15, 18–20). The last modality, even if still at its dawn, is elasto-sonography (ESG). ESG provides a mechanical characterization of the tissues, relying on the distortion obtained through a mechanical stimulus, which can be delivered by US impulses (quantitative representation- shear wave elastography) or by brain pulsation and probe motion (qualitative representation – strain elastography) (21). Interestingly, by assessing the mechanical properties of the tissues, it is possible to highlight most of the lesions and characterize their features such as degree and histotype (12, 21–27). Regardless of the employed modality, during the process of surgery, ioUS semiotics and image quality are subjected to modifications and deterioration. Different explanations have been proposed leading to specific adjuncts, such as mini-probes (28, 29), specific fluid to fill the cavity (30), contrast-enhanced ultrasound (19), and tangential trans-cortical scanning (5), which have further improved ioUS potential. We are still witnessing innovation and research in the field of ioUS applications in neurosurgery.

This Research Topic reports the experiences of leading experts in the field, presenting an updated portrait of the state-of-the-art and future perspectives of ioUS. Carai et al. present their experience on ioUS in pediatric neurosurgical oncology, where they observed a good predictive value on the extent of resection of ioUS in children. Šteňo et al. address ioUS from a different perspective by presenting on current limitations, such as suboptimal quality in some pathologies, different types of artifacts, patient positioning, and the unavailability of probes to depict the entire sellar region finally presenting valuable insights and solutions. Bastos et al. describe the use of 3D ioUS with intra-operative MRI, demonstrating that ioUS, in most cases, defines tumor location, tumor margins, relevant landmarks for orientation, and predicts the extent of resection. Incekara et al. performed a randomized controlled trial in glioblastoma patients (NCT03531333), demonstrating that ioUS (B-mode) leads to a complete resection in a higher number of patients if compared to standard surgery. Chan et al., relying on their experience with Shear Wave Elastography (SWE), observed a higher sensitivity of SWE and an improved ability to detect residual tumor, thus sustaining the combination of surgeon’s opinion and ioUS to enhance the extent of resection. Gueziri et al. present a novel US-based registration for spinal procedures which yields an acceptable accuracy if compared to CT-based procedures. Saß et al. present a novel approach to amend the brain shift in intra-axial tumor surgery. In particular, they developed a method based on 3D ioUS color Doppler to estimate the amount of brain shift intraoperatively, thus potentially allowing an update of the navigation system. Gerard et al. undertook a comprehensive review, dissecting the limitation and impact of brain-shift in neuro-navigation and focusing on ioUS-based technologies for measuring and compensating its occurrence. Different ioUS approaches are presented, together with the major unmet clinical need for newer openly available clinical datasets. Notably, ioUS would benefit not only from US technical improvements but also from emerging technologies such as machine learning and modern graphics processing units (GPUs), which strictly depend on the databases of ioUS images to train the algorithms. In this context, Reinertsen et al. present the current situation of the large number of projects that have profited from the publicly available datasets that include MR and US data from brain tumor cases, finally advocating for the realization of an organized platform to prospectively collect data to develop and validate machine learning algorithms. On this line, Cepeda et al. exploited an automated deep learning approach for image analysis. They demonstrate that the automated processing of ioUS through deep learning can generate high-precision algorithms to differentiate glioblastomas from metastases, in particular with strain elastography.

This collection of articles provides a wide overview of ioUS applications in neurosurgery, enhancing the level of evidence in favor of the use of ioUS in neurosurgical oncology, and shedding light on some promising research lines and unmet needs in this field.

## Author Contributions

MB, FD, and GU: manuscript concept and design. FD and GU: final revision. All authors contributed to the article and approved the submitted version.

## Conflict of Interest

The authors declare that the research was conducted in the absence of any commercial or financial relationships that could be construed as a potential conflict of interest.

## Publisher’s Note

All claims expressed in this article are solely those of the authors and do not necessarily represent those of their affiliated organizations, or those of the publisher, the editors and the reviewers. Any product that may be evaluated in this article, or claim that may be made by its manufacturer, is not guaranteed or endorsed by the publisher.
